# Assessing the Sensitivity of *Plasmopara halstedii* Isolates to Mefenoxam through Host Responses

**DOI:** 10.3390/microorganisms11040821

**Published:** 2023-03-23

**Authors:** Nisha Nisha, Sergey Vinogradov, Katalin Körösi, Arbnora Berisha, Rita Bán

**Affiliations:** 1 Department of Integrated Plant Protection, Institute of Plant Protection, Hungarian University of Agriculture and Life Sciences, H-2100 Godollo, Hungary; nisha27evs@gmail.com (N.N.); korosi.katalin.orsolya@uni-mate.hu (K.K.); a.berisha5@hotmail.com (A.B.); 2 Department of Agricultural Data Processing and Data Analysis, Institute of Agricultural and Food Economics, Hungarian University of Agriculture and Life Sciences, H-2100 Godollo, Hungary; vinogradov.szergej@uni-mate.hu

**Keywords:** sunflower downy mildew, fungicide resistance, fluorescence microscopy, host reactions, hypersensitive reaction, mefenoxam

## Abstract

Downy mildew caused by *Plasmopara halstedii* is responsible for significant economic losses in cultivated sunflowers. Field isolates of sunflower downy mildew resistant to mefenoxam, a previously effective active ingredient against the pathogen, have been found across Europe. The main goal of this study was to assess the sensitivity of *P. halstedii* isolates to mefenoxam through host responses to infection, such as symptoms measured by disease severity and growth reduction, and host tissue reactions, such as hypersensitive reaction and necrosis of invaded cells. Sunflower seeds were treated with Apron XL 350 FS at the European registered rate (3 mg/kg seeds). Seedlings were inoculated using the soil drench method with eight Hungarian *P. halstedii* isolates. Disease rates and plant heights were measured twice. Histological examinations of cross-sections of sunflower hypocotyls were performed using a fluorescence microscope. In our study, cluster analyses of sunflowers based on macroscopic and microscopic variables showed differentiation of groups of mefenoxam-treated sunflowers inoculated with different *P. halstedii* isolates. We first revealed a clear difference in host responses of mefenoxam-treated susceptible sunflowers. In addition, examining tissue reactions (e.g., hypersensitive reaction, necrosis) seems more accurate to estimate the sensitivity of *P. halstedii* isolates to mefenoxam than macroscopic symptoms.

## 1. Introduction

Sunflower (*Helianthus annuus* L.) is one of the essential crops in the world and the second most widely farmed oil seed in the European Union. Sunflower oil production worldwide was 19.2 million tons in 2020 [1]. Diseases can significantly compromise crop security by reducing yield and affecting oil content. For example, crop yield loss and quality degradation caused by plant pathogens can be up to 100% in sunflowers [2] (pp. 201–226). Sunflower downy mildew caused by the pathogen *Plasmopara halstedii* (Farl.) Berl. et de Toni is one of the most severe global diseases impacting production [3]. *Plasmopara halstedii* is an obligate biotrophic oomycete that needs a living host to complete its life cycle [4]. This pathogen is diploid and homothallic, able to reproduce asexually and sexually. Sunflower downy mildew spreads via wind and infected seeds; however, *P. halstedii* is mainly soil-borne [5]. The pathogen infects seedlings via their roots through zoospores, leading to systemic infection, and may cause local foliar lesions via airborne sporangia. Moreover, a local infection can turn systemic, resulting in the deformation of upper plant parts [6].

The symptoms of downy mildew in sunflower vary depending on the age of the tissue, the cultivars/genotypes utilized, and the environmental conditions at the time of infection [7]. Infected plants are underdeveloped and dwarfed, with chlorotic leaves coated with white sporangiophores and sporangia [8]. A high percentage of infection in the field, a short latent period, a high sporulation density, and a significant reduction in the hypocotyl length indicate the high aggressiveness of the pathogen [9]. Although severely diseased plants may die before or soon after emergence or during the seedling stage, most symptomatic plants survive but do not produce viable seeds. The potential yield loss after primary infection is often as high as 50% [2].

Aside from crop rotation, resistance breeding and chemical seed treatment are fundamental ways of controlling sunflower downy mildew [8]. Dominant *Pl* genes (downy mildew resistance genes) incorporated into sunflower hybrids confer resistance to the disease. However, several new virulent *P. halstedii* pathotypes have developed, overcoming the effect of those genes [10]. There are currently 50 pathotypes (virulence phenotypes) of the pathogen worldwide [3]. Regarding seed treatment, metalaxyl, a systemic phenyl amide, has been widely used to control different oomycetes because of its excellent preventive, curative, and eradicative effects [5]. Metalaxyl was later substituted with its stereoisomer, mefenoxam (metalaxyl-M), which is effective even at lower rates. Metalaxyl and mefenoxam are active ingredients of single-site fungicides that affect the specific metabolism of the target pathogen [11]. They block the rRNA biosynthesis (polymerase complex I) of pathogens, inhibiting mycelial growth and sporulation.

Shortly after the first field application of metalaxyl and mefenoxam, tolerant strains could be identified for several oomycetes (see Gisi and Sierotzki [12] (pp. 145–174) for review). First, Oros and Viranyi [13] showed resistance of *P. halstedii* to metalaxyl in greenhouse experiments in Hungary. Later, Delen et al. [14] also detected decreased pathogen sensitivity to this active ingredient in Turkey. Soon after, Lafon et al. [15] and Albourie et al. [16] in France, Gulya [17] (pp. 79–84) in the USA, Molinero-Ruiz et al. [18] in Spain, Körösi et al. [19] in Hungary, and Iwebor et al. [20] in Russia reported that some *P. halstedii* isolates were not controlled when the registered rate of mefenoxam was applied. The expression of fungicide resistance employed by the FRAC (Fungicide Resistance Action Committee) refers to an acquired, heritable reduction in the sensitivity of a pathogen to a particular fungicide [21]. The mechanism of resistance to mefenoxam has yet to be discovered. However, it has shown to be quantitative, i.e., the reduction in disease control and the loss of sensitivity of pathogen populations is gradual and partial. Furthermore, mefenoxam is an active ingredient with a high risk of resistance, according to the FRAC code list [22].

Since the causal agent of sunflower downy mildew is biotrophic, i.e., a living plant is necessary for its development, the sensitivity of *P. halstedii* isolates to mefenoxam can be measured through the plant’s response. Earlier studies have established the sensitivity of *P. halstedii* to mefenoxam mainly based on symptoms (dwarfing, leaf chlorosis) and signs (sporulation) on the infected plants. In addition, the pathogen’s development in the mefenoxam-treated susceptible seedlings was also studied with fluorescent microscopy by Mouzeyar et al. [23]. Similar host responses in the mefenoxam-treated plants to the pathogen, such as hypersensitive reactions, necrosis, and cell division, were found to be that of the genetically resistant, non-treated sunflowers. However, only a *P. halstedii* isolate sensitive to mefenoxam was included in that study. Furthermore, the studies that have tested the sensitivity of several *P. halstedii* isolates to mefenoxam are primarily in vivo tests examining macroscopic symptoms and signs of the treated and inoculated plants [16,17,19]. While understanding plant tissue responses to different pathogen variants is essential for safe crop production, the goals of this work were the following:

(1) to study the histopathology of hypocotyl infection in a susceptible sunflower cultivar inoculated with *P. halstedii* isolates with varying degrees of sensitivity to mefenoxam;
(2) to assess the sensitivity of *P. halstedii* isolates to mefenoxam through host responses to infection, such as symptoms measured by disease severity and growth reduction, and host tissue reactions, such as hypersensitive reaction and necrosis of invaded cells.

## 2. Materials and Methods

### 2.1. Plant Material and Treatment of Seeds with Mefenoxam

The sunflower cultivar Iregi szurke csikos was used in this experiment. It is susceptible to all pathotypes of *P. halstedii* because of the absence of *Pl* resistance genes. Seeds were disinfected via immersion in 1% sodium hypochlorite solution (42 g L^−1^ NaOCl) for 5 min and then rinsed with running tap water. Seeds were treated with Apron XL 350 FS (350 g/L mefenoxam, Syngenta AG, Switzerland) as per the European registered rate, i.e., 3 mg *a.i.* kg^−1^ seeds (*a.i.* = active ingredient) were evenly coated with the fungicide by mixing in a beaker. Treated seeds were kept for drying at room temperature for three days, then planted in pots (d = 8 cm, depth of sowing: 1.5 cm) containing clean, moistened perlite and, except for the sporulation induction period (for 24 h at 19 °C), kept in a growth chamber for 21 days (22 °C, relative humidity: 70%, 12 h photoperiod, light irradiance of 100 µE·m^−2^ ·s ^−1^).

### 2.2. Experimental Design

Mefenoxam-treated inoculated and non-treated inoculated seeds were placed in pots (5 seeds per pot) and arranged in trays (10 pots per tray) for each *P. halstedii* isolate. Mefenoxam-treated non-inoculated and non-treated non-inoculated controls were also treated with the same arrangement as the inoculated ones to check normal plant growth. The experiment was repeated twice.

### 2.3. Plasmopara halstedii Isolates, Preparation of Inoculum, and Inoculation

Eight *P. halstedii* isolates collected in different years and locations were selected for this study from the collection of the Department of Integrated Plant Protection (Institute of Plant Protection, MATE, Godollo, Hungary) (Table 1). Isolates were stored at −70 °C on infected leaves in plastic Petri dishes. Pathotype identification of these isolates was made previously as a part of a survey between 2012 and 2019 in Hungary [3].

During inoculum preparation, frozen leaves with sporangia of *P. halstedii* were washed off in bidistilled water. The suspension concentration was adjusted to 50,000 sporangia per ml using a Bürker counting chamber. Three days after sowing, seedlings were inoculated using the soil drench method [24], i.e., the sporangial suspension (2 mL per seedling) was pipetted directly onto the perlite surface of each pot containing the seedlings. Non-inoculated control plants were included to ensure the damping-off symptom was attributable to the disease. For non-inoculated plants, bidistilled water was drenched over seedlings. Then, plants were kept at 16 °C in the dark for 24 h to ensure infection.

### 2.4. Disease Assessment 

Nine days after inoculation, plants were sprayed with bidistilled water and covered with a dark polyethylene bag. Then, pots were placed in the dark for 24 h at 19 °C (relative humidity: 90–100%) to induce sporulation. The first evaluation was based on white coating (sporangiophores and sporangia) on cotyledons and pre-emergence damping-off, referring to Disease 1. Twenty-one days after inoculation, a second evaluation was made according to chlorosis along the veins of the true leaves, if they had them, or post-emergence damping-off, referring to Disease 2. Disease rates (%) for Disease 1 and 2 values were calculated as the percentage of diseased plants for all isolates. Plant heights were measured during each disease assessment (Height 1 and 2).

### 2.5. Microscopic Observations

Histological examinations of cross-sections of sunflower hypocotyls were performed using a fluorescence microscope (Olympus, Japan; filter block BX 50, transmission > 515 nm). Twenty-one days after inoculation, five sunflower hypocotyls were selected from each treatment (treated and non-treated with mefenoxam and inoculated with different *P. halstedii* isolates, respectively) and fixed in FAA solution (formalin: acetic acid: ethanol, 10:5:50 by vol.). Then, thin cross-sections (15–20 pieces) were cut with a razor blade from both the upper and lower parts of the hypocotyl and examined for pathogen structures (hyphae, haustoria) and tissue responses (hypersensitive reaction, cell necrosis). The hypersensitive reaction was defined by the autofluorescence of the cells and necrosis by the presence of brown, dead cells. In accordance with Bán et al. [25] (pp. 265–273), a 0–4 scale was used to observe pathogen structures and host responses. Briefly, the sections were divided theoretically into four quarters (both the cortical and pith parenchyma), and the presence of the pathogen and the plant responses were examined in each.

### 2.6. Statistical Analysis

Differences in disease rates, host characteristics (plant height), and host responses (HR and cell necrosis) were assessed by the one-way analysis of variance (ANOVA) followed by the Tukey HSD (honestly significant difference) multiple comparison post hoc test. In addition, the Kolmogorov–Smirnov test was used to test the normality of the distribution of the data within groups. Levene’s test was used to determine whether variances were equal.

Two-way ANOVA was used to examine the interaction between treatment (non-treated, treated) and isolates. Using Ward’s method, hierarchical cluster analysis was performed to group *P. halstedii* isolates based on their sensitivity to mefenoxam. To examine the correlation between variables, Pearson’s correlation coefficient was performed for scale variables (disease rates, heights), and Spearman’s correlation coefficient was used for ordinal variables (microscopic variables). IBM SPSS Statistics 27 software was used to conduct the statistical analysis.

## 3. Results

### 3.1. Disease Rates and Heights

**Figure 1:**
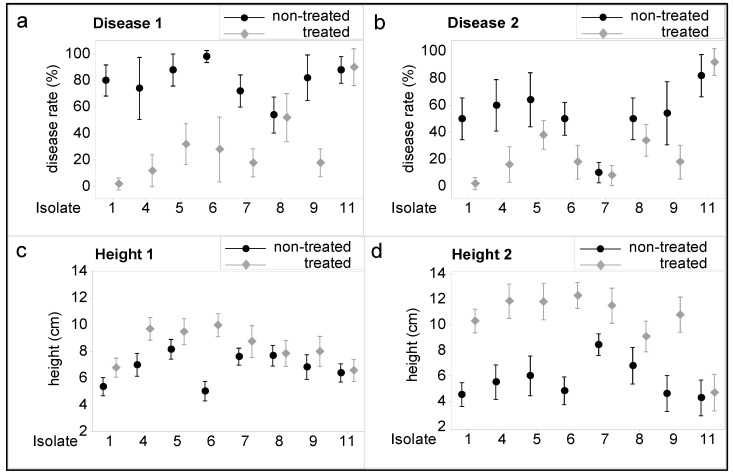
Disease rates (**a**,**b**) and heights (**c**,**d**) of mefenoxam-treated and non-treated sunflower plants inoculated with different *Plasmopara halstedii* isolates. Disease 1: ratio of sporulating pre-emergence damped-off plants and healthy sunflowers nine days after inoculation. Disease 2: ratio of chlorotic post-emergence damped-off plants and healthy sunflowers 21 days after inoculation. Height 1: height of sunflowers nine days after inoculation (heights of damped-off plants were taken as zero). Height 2: height of sunflowers 21 days after inoculation (heights of damped-off plants were taken as zero). Treatment: non-treated and treated with mefenoxam (3 mg/kg seed). Isolate: code of *Plasmopara halstedii* isolates used in the experiment (1, 4, 5, 6, 7, 8, 9, and 11) (for more details, see Table 1). Vertical lines represent 95% confidence intervals (95% CI) of the mean values of disease rates and heights.

Disease rates (%) and heights of mefenoxam-treated and non-treated sunflower plants inoculated with different *P. halstedii* isolates are shown in Figure 1. According to the sporulation of the pathogen on the cotyledons and pre-emergence damped-off plants (Disease 1, Figure 1a), mefenoxam-treated sunflowers inoculated with isolates 1, 4, 5, 6, 7, and 9 showed significantly lower infection rates compared to non-treated ones. However, there were no significant differences in disease rates between treated and non-treated plants inoculated with isolates 8 and 11. The situation was similar with Disease 2 (ratio of chlorotic post-emergence damped-off plants and healthy sunflowers, Figure 1b), but there was no difference in the disease rate of treated and non-treated plants inoculated with isolates 7 in addition to isolates 8 and 11.

Plant heights of mefenoxam-treated sunflowers inoculated with *P. halstedii* isolates 1, 4, 5, and 6 were significantly higher than those of the non-treated inoculated plants nine days after inoculation (Figure 1c). On the contrary, there was no significant difference in plant heights between treated and non-treated sunflowers inoculated with isolates 7, 8, 9, and 11. However, by the second recording date, the height of the treated plants was significantly higher than that of the non-treated plants for all isolates except 11 (Figure 1d).

For all parameters tested (Disease 1–2, Height 1–2), the interaction between isolate and treatment was significant (for Disease 1: F = 12.06, *p* < 0.001, for Disease 2: F = 5.36, *p* < 0.001, for Height 1: F = 6.61, *p* < 0.001, for Height 2: F = 7.37, *p* < 0.001), i.e., the impact of treatment varied between isolates.

### 3.2. Microscopic Observations

**Figure 2:**
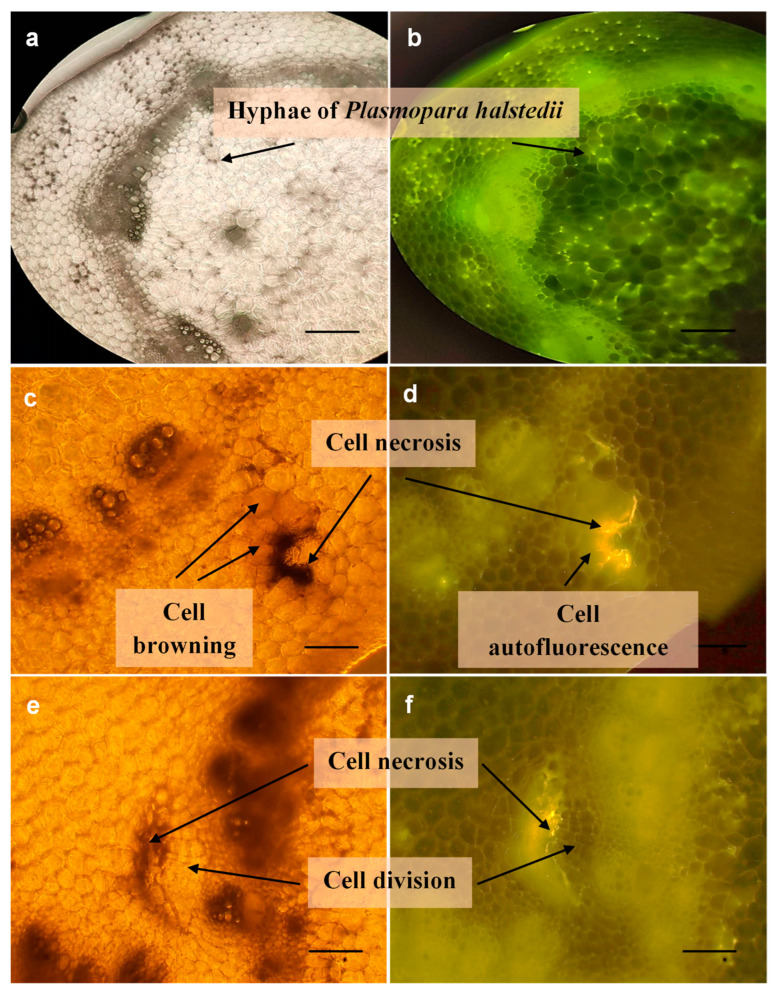
Light micrographs of mefenoxam-activated resistance responses in hypocotyl cross-sections of sunflower. Hyphae of *Plasmopara halstedii* invade cells of non-treated, inoculated susceptible plants (cv. Iregi szürke csíkos) without any host responses in normal (**a**) and in UV light (**b**) (λ = 485 nm) at 21 dpi. Browning (**c**), autofluorescence (hypersensitive reaction) (**d**), and necrosis with intensive cell division (**e**: normal light, **f**: UV light) of cortical parenchyma cells neighboring invaded cells as a host response to the pathogenic attack of mefenoxam-treated inoculated plants at 21 dpi. Scale bar = 100 µm.

Sunflower tissue responses to infection by *P. halstedii* in hypocotyl cross-sections are shown in Figure 2. Similar tissue responses were observed in most treated and non-treated plants infected with different isolates, but the intensity of the pathogenic spread and tissue responses were variable (see below). In general, intercellular hyphae and intracellular haustoria were detected in the hypocotyl of non-treated plants in the cortical and the pith parenchyma 21 days after inoculation (Figure 2a). Under UV light, autofluorescence appeared in the intercellular spaces around hyphae, giving the image a dotted appearance (Figure 2b). In contrast, cell browning under normal light (Figure 2c) and an intense autofluorescence of cells showing a hypersensitive-like reaction (Figure 2d) could be detected in cross-sections of several mefenoxam-treated and inoculated sunflowers. Moreover, the development of cellular necrosis by vigorous cell division (Figure 2e) and the strong fluorescent response of surrounding cells (Figure 2f) was also frequently observed in treated and inoculated plants.

**Figure 3:**
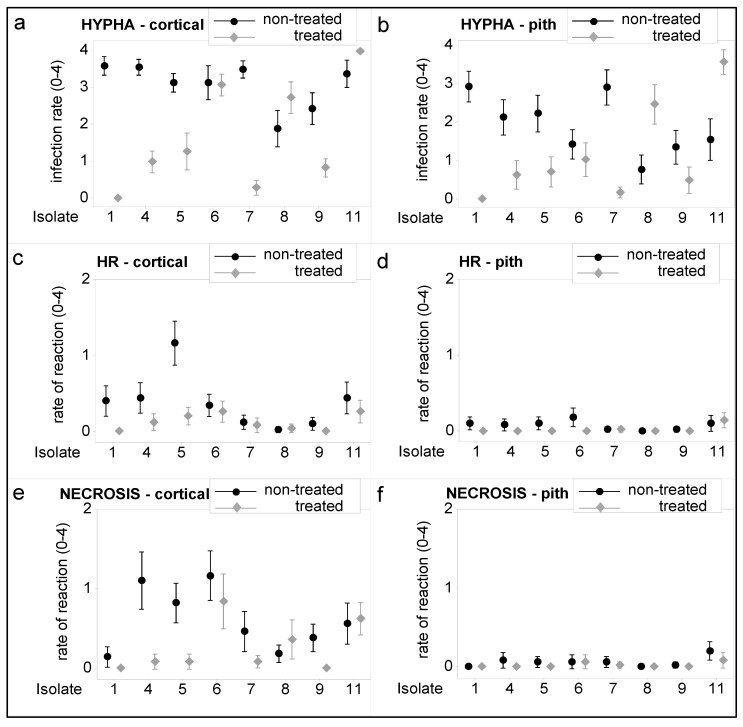
Occurrence of pathogen hyphae (**a**,**b**) and host reactions such as hypersensitive reaction (**c**,**d**) and necrosis (**e**,**f**) in the cortical and pith parenchyma of mefenoxam-treated and non-treated sunflower plants inoculated with *Plasmopara halstedii*. Treatment: non-treated and treated with mefenoxam (3 mg/kg seed). Isolate: code of *Plasmopara halstedii* isolates used in the experiment (1, 4, 5, 6, 7, 8, 9, and 11) (for more details, see Table 1). The infection rate and the rate of the host reaction were measured on a 0–4 scale. Vertical lines represent 95% confidence intervals (95% CI) of the mean values of disease rates and heights.

The rates of pathogen hyphal spread and tissue responses are shown in Figure 3. Hyphae spread significantly in the cortical and pith parenchyma of non-treated plants inoculated with isolates 1, 4, 5, and 7 compared to mefenoxam-treated plants (Figure 3a,b). In contrast, more hyphae were found in the cortical and pith parts of mefenoxam-treated sunflowers inoculated with *P. halstedii* isolate 8 than in non-treated ones. The situation was similar to the appearance of hyphae of isolate 11 in the pith. In addition, hyphae were significantly more abundant in the cortical part of non-treated sunflowers inoculated with isolate 9, whereas there was no significant difference in hyphal distribution between treated and non-treated sunflowers for isolate 6 (Figure 3a,b).

Generally, fluorescence microscopy of cross-sections of sunflower hypocotyls revealed a relatively higher rate of hypersensitive-like reaction and necrosis (cell death) in the cortical than in the pith parenchyma in this experiment (Figure 3c–f). The hypersensitive reaction was prominent in non-treated plants inoculated with isolate 5 and to a smaller extent in non-treated sunflowers inoculated with isolates 1, 4, 6, and 11 in the cortical parenchyma (Figure 3c,d). However, it was not significant for the latter two compared to mefenoxam-treated plants. The occurrence of cell necrosis in the cortical part was intensive in non-treated plants inoculated with isolates 4, 5, and 6. The latter was not significant compared to mefenoxam-treated sunflowers (Figure 3e). Necrosis in the pith parenchyma cells was minimal in each sample (Figure 3f).

### 3.3. Assessing the Sensitivity of Plasmopara halstedii Isolates to Mefenoxam

Cluster analyses of sunflowers inoculated with different *P. halstedii* isolates based on disease rates and plant heights are shown in Table 2. Four distinct clusters could be identified using macroscopic parameters. Cluster 1 includes non-treated plant samples inoculated with isolates 5, 6, 9, and 11, and mefenoxam-treated plants inoculated with isolate 11, which were found to have high infection levels in both sampling periods. Therefore, the pathogen could penetrate the upper parts of these sunflowers. Plant heights were the lowest in this group. In Cluster 2 are samples of the other parts of non-treated and inoculated plants, where the first infection value (Disease 1) was relatively high, as in Cluster 1. However, unlike the first cluster, the second time point for disease assessment (Disease 2) resulted in much lower infection values and less plant dwarfing in Cluster 2 members (Table 2). In this case, the pathogen could only penetrate to a lesser extent above the hypocotyl. 

Clusters 3 and 4 mainly include samples of inoculated plants treated with mefenoxam. In contrast to the initial infection rates, there was no significant difference between the two clusters in the second survey. However, the plant height values were significantly higher for Cluster 3 members (Table 2).

Cluster analyses of sunflowers based on the examined microscopic variables inoculated with different *P. halstedii* isolates are presented in Table 3. Three distinct clusters could be identified using microscopic parameters. Samples of non-treated inoculated plants are in the first two clusters, while mefenoxam-treated plants can be found in all three clusters. Moreover, treated plants inoculated with isolates 4 and 5 are equally represented in the first two clusters.

For Cluster 1 samples, the pathogen could invade both the cortical and pith parenchyma (Table 3). Not only the spread of hyphae but also the HR and necrosis in different tissue sections were significant in Cluster 1 samples compared to the other two clusters. Treated sunflowers inoculated with *P. halstedii* isolates 8 and 11 are included in the first cluster along with non-treated ones. Unlike the sunflowers in the first cluster, the distribution of hyphae of samples in Cluster 2 (isolates 1, 4, 7, and 8, non-treated) was accompanied by HR and necrosis only in the cortical parenchyma but not in the pith. Most of the treated sunflower samples, except for isolates 6, 8, and 11, are in Cluster 3 (isolates 1, 4, 5, 6, 7, and 9, treated), with few hyphae detected in the cortical tissues. No tissue response was detected in these sunflowers.

### 3.4. Correlations among Macroscopic Parameters

The results of Pearson correlation based on the examined macroscopic variables (disease rates, plant heights) are shown in Table 4. During the second evaluation, a strong negative correlation was found between the disease rate and plant height values of both non-treated and treated plants. Similarly, the experiment showed a strong negative correlation between the initial disease rates and the final plant height values of treated plants. In contrast, a high positive correlation could be detected between the initial and final plant height data of both treated and non-treated plants. In addition, a strong positive correlation was found between the initial and final disease values of mefenoxam-treated sunflowers.

### 3.5. Correlations among Microscopic Parameters

The Spearman correlation of the examined microscopic variables is presented in Table 5. There was a strong positive correlation in the occurrence of hyphae in different parenchymatic plant parts (cortical and pith) of both non-treated and treated inoculated sunflowers. Moreover, strong positive correlations were found between the presence of hyphae in the cortical parenchyma tissues and the appearance of hypersensitive reaction and necrosis in treated plants. In addition, a strong positive correlation could be confirmed between the establishment of necrosis in the cortical part and the occurrence of hyphae in the pith of mefenoxam-treated and inoculated sunflowers.

## 4. Discussion

Field isolates of sunflower downy mildew resistant to mefenoxam, a previously effective active ingredient against the pathogen, were found across Europe [15,16,18,20] and in the USA [17] (pp. 79–84). There are no data from Asia and Africa on mefenoxam resistance in the pathogen. Moreover, in a recent study, seven out of ten *P. halstedii* isolates collected in Hungary showed poor to moderate sensitivity to mefenoxam [19]. According to our present study, with more detailed symptom recording and refined statistical analyses than in previous studies, reduced sensitivity could be measured for three out of eight downy mildew isolates.

Cluster analysis based on disease rates and plant heights showed a difference between mefenoxam-treated and non-treated plants in this experiment. The only exception was sunflowers inoculated with *P. halstedii* isolate 11, where the values of mefenoxam-treated and non-treated plants were similar to those of other non-treated plants. In addition, both treated and non-treated plants formed two relatively distinct groups (clusters) based on cluster analysis of disease rates and plant heights. The sunflowers in Cluster 1 (non-treated and inoculated with isolates 5, 6, 9, and 11) had relatively high initial and subsequent infection rates, indicating that the pathogen could penetrate unhindered into the upper parts of the plant. This was associated with significant growth inhibition of these plants. On the other hand, the reaction was similar in mefenoxam-treated plants inoculated with isolate 11; this *P. halstedii* isolate therefore appears to be mefenoxam resistant. Interestingly, in Cluster 2, non-treated plants inoculated with isolates 1, 4, 7, and 8 were characterized by decreased spreading of the pathogen to the aboveground plant parts compared to Cluster 1. The difference in pathogen spread between the two clusters of mainly non-treated plants is likely explained by the different aggressiveness of the *P. halstedii* isolates tested, a common phenomenon indicated by other authors [9].

Nevertheless, the two clusters of mefenoxam-treated and inoculated plants (Clusters 3 and 4) also differed, mainly in the degree of initial disease rate and the development of plant heights. In conclusion, treatment with mefenoxam had different effects on different *P. halstedii* isolates, according to disease rates and plant heights.

Pearson correlation, especially during the second evaluation, showed a strong negative correlation between the disease rate and plant height values of both non-treated and treated plants. This negative correlation is not surprising, as many authors have reported such effects of the pathogen on plant development in susceptible, non-treated sunflowers [8,26]. In the case of treated plants, this negative correlation is presumably related to fungicide resistance since if the pathogen can spread within the plant, the growth-reducing effect is exerted.

The main objective of this study was to investigate the tissue responses of treated plants inoculated with different *P. halstedii* isolates with a fluorescent microscope. Host responses of sunflowers (susceptible, resistant) inoculated with *P. halstedii* have already been examined by several authors [25,27,28,29,30,31,32]. Mouzeyar et al. [30,31] pointed out that *P. halstedii* could infect susceptible and resistant sunflower lines in a microscopic investigation, although to a lesser extent, even a susceptible plant can react to the pathogen’s growth. Our results with fluorescent microscopy of non-treated sunflowers also supported this (Figure 3c–f, Table 3). Mefenoxam treatment alone did not induce autofluorescence in the treated plants in our studies, it was only when the treated plants were inoculated with the pathogen. Autofluorescence, one of the tissue reactions during host–parasite interactions, is mainly associated with the appearance of phenolic compounds (e.g., phytoalexins, lignin), which play an essential role in the plant’s defense processes against the pathogen [30].

Moreover, the speed and intensity of host response to *P. halstedii* in a resistant sunflower may vary, and it can appear in the root or different parts of the hypocotyl [30]. Previous authors also described a hypersensitive-like response in the hypocotyl of mefenoxam-treated susceptible sunflowers [23]. They found that all metalaxyl concentrations and application modes provided complete protection against *P. halstedii*. However, only one *P. halstedii* isolate was tested in the latter work that seemed sensitive to the active ingredient.

We first revealed a clear difference in host responses of mefenoxam-treated susceptible sunflowers inoculated with various *P. halstedii* isolates. Treated plants inoculated with some isolates (6, 8, and 11) showed hyphal growth in the cortical and pith parenchyma. A moderate hypersensitive reaction and necrosis could also be detected in the cortical part. This phenomenon was very similar to what usually occurs in non-treated susceptible plants, with the plant response appearing to be a delayed host reaction to a pathogenic attack [8,30]. For other *P. halstedii* isolates, we could detect limited or no mycelial growth in the mefenoxam-treated plants, which was accompanied by a weak or no reaction of the treated sunflowers in their hypocotyls. Because of the lack of massive mycelial growth in the hypocotyl, it is likely that the pathogen was arrested in the root tissues by the chemical.

In our study, cluster analyses of sunflowers based on microscopic variables showed clear differentiation of three groups of mefenoxam-treated sunflowers inoculated with different *P. halstedii* isolates. Those in the first two groups (clusters) showed high (isolates 8 and 11) or moderate resistance (isolate 6) to mefenoxam, while isolates in the third group showed sensitivity. Disease rate and plant height values (macroscopic parameters) of treated and inoculated sunflowers with these resistant isolates also supported this (Table 3). However, only isolate 11 could be defined as having highly decreased sensitivity with the evaluation of visible symptoms (macroscopic parameters) (Figure 3). Hence, examining tissue reactions (e.g., hypersensitive reaction, necrosis) seems more accurate for estimating the sensitivity of *P. halstedii* isolates to mefenoxam than macroscopic symptoms.

In addition to its direct toxic effect on the pathogen, metalaxyl activates the host defense system, which might result in increased sunflower resistance, restricting pathogen development [33,34]. In previous research, histological alterations such as haustoria encapsulation by callose deposits [35] or the development of limited hypersensitive-like lesions were also reported, followed by metalaxyl treatment in some host–parasite interactions where the pathogen was sensitive to the chemical [23,36,37,38]. However, the question remains whether the direct (fungistatic) or indirect effect (through the host) of metalaxyl is more significant against the sensitive pathogen in different host–parasite relationships.

Examining metalaxyl-sensitive and tolerant *Phytophthora megasperma* isolates in soybean, Cahill and Ward [39] pointed out that metalaxyl enhanced the release of phytoalexin elicitors (glyceollin) in culture fluids of the sensitive isolate but not in those of the tolerant isolate. Releasing elicitors due to metalaxyl treatment could induce host reactions in compatible interactions with the sensitive isolate. In our study, the effective host responses against the sensitive P. halstedii isolates likely occurred at a very early stage of infection in the roots of mefenoxam-treated sunflowers. Despite this, the reaction of mefenoxam-treated plants to resistant isolates could appear later in the hypocotyl, which the delayed stimulation of elicitor activity by the chemical can explain. Our results with the Spearman correlation also demonstrate this. It showed that the spread of the resistant isolates in the cortical parenchyma of treated plants correlated positively with the appearance of HR and necrosis.

Interestingly, more abundant hyphae were found in the pith of treated than non-treated plants inoculated with isolates 8 and 11 (considered resistant). This is in line with the results of Cahill and Ward [39], who reported better growth of metalaxyl-tolerant *Phytophthora megasperma* isolates in the presence of the chemical in vitro and in vivo. Previous authors assumed that metalaxyl could serve as a nutrient and raised the idea of other resistance mechanisms and different interactions with the host (soybean) for those tolerant isolates. In addition, the more significant presence of *P. halstedii* in the pith of sunflowers has been shown to facilitate the spread of the pathogen to the upper parts of the plant (e.g., epicotyl) [40].

Further studies are needed to explore the reasons for the differences in tissue responses to sensitive and resistant isolates of *P. halstedii* in sunflower. In addition, how plant defense mechanisms contribute to the effectiveness of fungicides also has to be elucidated.

## Figures and Tables

**Table 1:** List of *Plasmopara halstedii* isolates collected in Hungary (2012–2017) used in the experiment.

Isolate Code	Locality (County)	Year of Collection	Pathotype (Virulence Phenotype)
1	Mezőkovácsháza (Békés)	2017	724
4	Kömlő (Heves)	2014	704
5	Doboz (Békés)	2014	704
6	Körösladány (Békés)	2014	714
7	Szeghalom (Békés)	2017	724
8	Pély (Heves)	2017	704
9	Bonyhád (Tolna)	2017	724
11	Rákóczifalva (Jász-Nagykun-Szolnok)	2012	704

**Table 2:** Cluster analyses of sunflowers inoculated with different *P. halstedii* isolates based on disease rates and plant heights.

Variables	Cluster 1	Cluster 2	Cluster 3	Cluster 4
Disease 1 (%)	90.2 ± 6.9 d	72.2 ± 12 c	20.4 ± 12.3 a	38.2 ± 13.3 b
Disease 2 (%)	74.5 ± 10.8 c	29.6 ± 10.4 b	15.9 ± 8.6 a	27.3 ± 10.2 ab
Height 1 (cm)	6.0 ± 0.8 a	7.1 ± 0.4 b	9.7 ± 0.8 c	7.4 ± 0.6 b
Height 2 (cm)	4.0 ± 1.0 a	7.5 ± 0.6 b	11.7 ± 1.1 d	9.3 ± 0.9 c

Data represent the means of variables for each cluster. Values followed by means represent standard deviation. Different letters (a, b, c, d) indicate significant differences based on the Tukey HSD post-hoc test (*p* < 0.05) among clusters, but not comparable between variables. Cluster 1: isolates 1, **5, 6, 9, 11** non-treated, **11** treated. Cluster 2: isolates **1**, **4**, **7**, **8** non-treated. Cluster 3: isolates **1, 4, 5, 6**, 7, 9 treated. Cluster 4: isolates 1, 4, 5, 6, **7, 8, 9** treated, 8, 9 non-treated. Bold isolate numbers indicate dominance of that isolate in that cluster compared to other clusters.

**Table 3:** Cluster analyses of sunflowers inoculated with different *P. halstedii* isolates based on the examined microscopic variables.

Variables	Cluster 1	Cluster 2	Cluster 3
H_Cort	3.7 ± 0.3 c	3.0 ± 0.5 b	0.2 ± 0.2 a
HR_Cort	0.4 ± 0.4 c	0.2 ± 0.2 b	0 a
NEC_Cort	0.7 ± 0.5 c	0.5 ± 0.4 b	0 a
H_Pith	3.6 ± 0.4 c	0.5 ± 0.3 b	0 a
HR_Pith	0.1 ± 0.2 b	0 a	0 a
NEC_Pith	0.1 ± 0.2 b	0 a	0 a

Data represent the means of variables for each cluster. Values followed by means represent standard deviation. Different letters (e.g., a, b) indicate significant differences based on the Tukey HSD post-hoc test (*p* < 0.05) among clusters, but not comparable between variables. Cluster 1: isolates **1, 4, 5, 7** non-treated, **8, 11** treated. Cluster 2: isolates **4, 5, 6, 8, 9, 11** non-treated, **6** treated. Cluster 3: isolates **1, 4, 5, 7, 9** treated. Bold isolate numbers indicate dominance of that isolate in that cluster compared to other clusters. The underlined isolates were equally represented in the clusters concerned.

**Table 4:** Pearson correlation among the examined variables (disease rates, plant heights).

Variable	Disease 1	Disease 2	Height 1	Height 2
*Panel A: Non-treated (n = 80)*				
Disease 1	1	0.346 **	−0.465 **	−0.550 **
Disease 2		1	−0.439 **	−0.713 **
Height 1			1	0.737 **
Height 2				1
*Panel B: Treated (n = 80)*				
Disease 1	1	0.701 **	−0.368 **	−0.700 **
Disease 2		1	−0.329 **	−0.722 **
Height 1			1	0.741 **
Height 2				1

Disease 1: ratio of sporulating damped-off plants and healthy sunflowers nine days after inoculation. Disease 2: ratio of chlorotic damped-off plants and healthy sunflowers 21 days after inoculation. Height 1: height of sunflowers nine days after inoculation (heights of damped-off plants were taken as zero). Height 2: height of sunflowers 21 days after inoculation (heights of damped-off plants were taken as zero). Treatment: non-treated and treated with mefenoxam (3 mg/kg seed). ** Correlation is significant at the 0.01 level (2-tailed). Values in bold indicate a strong correlation between variables.

**Table 5:** Spearman correlation among the examined microscopic variables.

Variable	H_Cort	HR_Cort	NEC_Cort	H_Pith	HR_Pith	NEC_Pith
*Panel A: Non-treated (n = 200)*						
H_Cort	1	0.211 **	0.291 **	0.508 **	0.158 **	0.150 **
HR_Cort		1	0.240 **	0.193 **	0.375 **	0.080
Nec_Cort			1	0.223 **	0.155 **	0.172 **
H_Pith				1	0.156 **	0.248 **
HR_Pith					1	0.106 *
Nec_Pith						1
*Panel B: Treated (n = 200)*						
H_Cort	1	0.327 **	0.488 **	0.759 **	0.174 **	0.153 **
HR_Cort		1	0.072	0.213 **	0.241 **	0.029
Nec_Cort			1	0.547 **	0.079	0.180 **
H_Pith				1	0.204 **	0.169 **
HR_Pith					1	0.129 *
Nec_Pith						1

H: hyphae of Plasmopara halstedii, HR: hypersensitive reaction of invaded cells, Nec: necrosis, Cort: cortical parenchyma, Pith: pith parenchyma. ** Correlation is significant at the 0.01 level (2-tailed). * Correlation is significant at the 0.05 level (2-tailed). Values in bold indicate a strong correlation between variables.

## Data Availability

All data generated or analyzed during this study are included in this article.
